# Feedback loop centered on MAF1 reduces blood–brain barrier damage in sepsis-associated encephalopathy

**DOI:** 10.1186/s11658-025-00686-x

**Published:** 2025-01-20

**Authors:** Xuebiao Wei, Wenqiang Jiang, Zhonghua Wang, Yichen Li, Yuanwen Jing, Yongli Han, Linqiang Huang, Shenglong Chen

**Affiliations:** 1 Department of Geriatric Intensive Medicine, Guangdong Provincial Geriatrics Institute, Guangdong Provincial People’s Hospital, Guangdong Academy of Medical Sciences, Southern Medical University, 106, Zhongshan Er Road, Guangzhou , 510080 Guangdong China; 2 Department of Critical Care Medicine, Guangdong Provincial People’s Hospital, Guangdong Academy of Medical Sciences, Southern Medical University, Guangzhou, China

**Keywords:** Sepsis, Blood–brain barrier, MAF1, Ubiquitination, Apoptosis

## Abstract

**Background:**

A previous study found that MAF1 homolog, a negative regulator of RNA polymerase III (*MAF1*), protects the blood–brain barrier (BBB) in sepsis-associated encephalopathy (SAE); however, the related molecular mechanisms remain unclear.

**Subjects and methods:**

In this study, a rat sepsis model was constructed using the cecum ligation and puncture (CLP) method. In vitro, rat brain microvascular endothelial cells and astrocytes were stimulated with serum from the sepsis model rats. The loss of MAF1 protein levels and the molecular mechanisms leading to cell damage were investigated.

**Results:**

It was shown in the SAE models that *MAF1* was expressed at low levels. Knockdown of Cullin 2 (*CUL2*) stimulated the accumulation of MAF1 protein, attenuated the RNA sensor RIG-I/interferon regulatory factor 3 (IRF3) signaling pathway, and reduced cell apoptosis. Furthermore, it increased phosphatase and tensin homolog (*PTEN*) expression and inactivated the serine/threonine kinase (AKT)/mechanistic target of the rapamycin kinase (mTOR) signaling pathway. Interference with forkhead box O1 (*FOXO1*) inhibited *MAF1* expression and activated the RIG-I/IRF3 signaling pathway, while *MAF1* overexpression promoted *PTEN* expression, decreased cell apoptosis, and normalized autophagy.

**Conclusions:**

These findings demonstrate that *CUL2* promoted MAF1 ubiquitination and caused BBB injury in SAE. Through the regulatory loop of PTEN/AKT/FOXO1/MAF1, *CUL2* initiated the gradual downregulation of *MAF1*, which subsequently regulated polymerase III (Pol III)-dependent transcription and played essential roles in cell apoptosis in SAE.

*Clinical trial number*: not applicable.

**Graphical Abstract:**

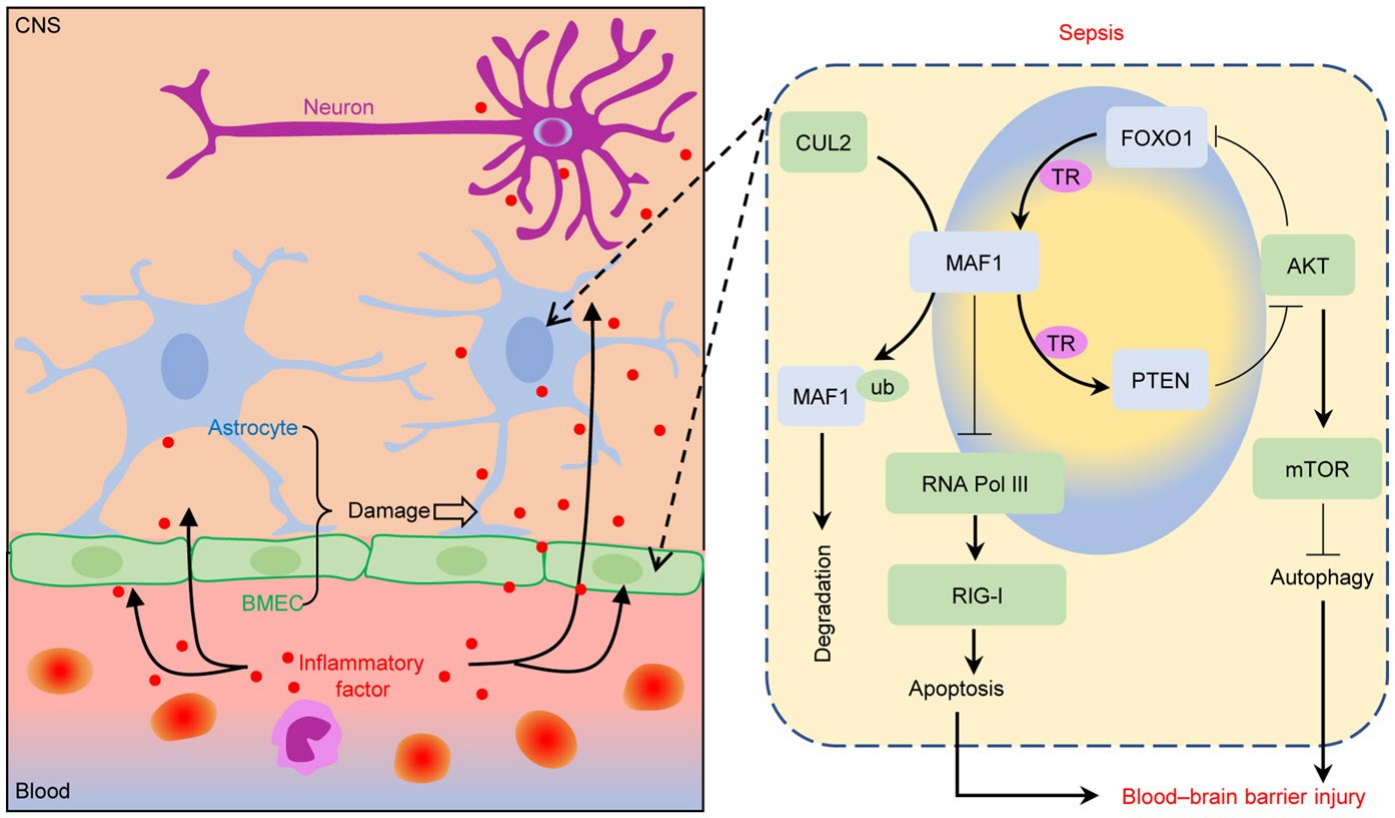

**Supplementary Information:**

The online version contains supplementary material available at 10.1186/s11658-025-00686-x.

## Background

Sepsis is a syndrome resulting from systemic inflammatory responses to infections, burns, shock, and surgery. Early sepsis can progress to severe sepsis that causes septic shock, resulting in multiple organ dysfunction syndrome and life-threatening low blood pressure [1]. More than 19.4 million cases of sepsis are diagnosed each year, and 5.3 million patients die from sepsis, with > 50% of deaths occurring in patients with severe sepsis [2]. SAE is a common complication of sepsis and is induced by impaired endothelial barrier function, excessive microglial activation, and blood–brain barrier (BBB) dysfunction [3, 4]. In recent years, numerous molecular mechanism studies have been performed on the BBB to investigate its unique role in regulating the progression of acute and chronic brain dysfunction [5, 6].

MAF1 homolog, a negative RNA polymerase III (*MAF1*) regulator, is a crucial repressor of RNA polymerase III (Pol III)-dependent transcription and function [7]. In a previous study, we found that *MAF1* was abnormally expressed in an in vitro model of BBB disruption and an in vivo model of SAE induced by lipopolysaccharide [8]. Furthermore, the regulation of *MAF1* can ameliorate SAE by suppressing the nuclear factor kappa B (NF-kB)/NLR family, pyrin domain containing 3 (*NLRP3*) inflammasome signaling pathway. Moreover, other scholars have reported that Pol III can promote caspase-3 mature shear and, at the same time, also encourage cell interferon production by activating downstream retinoic acid-induced gene I (*RIG-I*) and interferon regulatory factor 3 (*IRF3*), eventually activating inflammasomes [9, 10]. In addition, IFNβ signaling activation was mediated by RIG-I/IRF3 [11], which functions as a key role in sepsis [12]. However, its expression regulation and the molecular mechanism by which *MAF1* regulates BBB damage in patients with SAE remains unclear.

MAF1 is a labile protein, and its levels are regulated by the ubiquitin-dependent proteasome pathway. Wang et al. [13] reported that MAF1 ubiquitination can be promoted by mTOR complex 1 (TORC1)-mediated phosphorylation and critically regulated by E3 ubiquitin ligase Cullin 2 (*CUL2*). Regulation of *CUL2* can alter the stability of MAF1 and regulate Pol III-dependent transcription, resulting in sensitivity to doxorubicin-induced apoptosis. Ubiquitination is an important process for regulating protein translation [14]. As a member of the largest E3 ubiquitin ligase enzyme family, the Cullin-ring class E3 ubiquitin ligase enzyme is widely involved in regulating the degradation of cell cycle-related proteins and transcription factors in the body [15, 16]. CUL2 forms a ubiquitin ligase enzyme complex as a skeleton molecule and plays essential roles in tumor blood vessel production, cellular activity, immune escape, cell proliferation, and tumor malignant behavior [17]. For example, CUL2 was reported to be a component of the ElonginB/C-CUL2-RBX-1-Von Hippel-Lindau (VHL) tumor suppressor complex that ubiquitinates and degrades hypoxia-inducible factor α (HIFα) [18]. Wang et al. [19] demonstrated that CUL2 reduces the stability of PRDM16 protein, and thus assists in regulating glucose intolerance, diet-induced obesity, insulin resistance, and dyslipidemia in mice. However, whether and how MAF1 ubiquitination regulates BBB cell apoptosis via CUL2 remains unknown.

Another possible regulatory pathway is modulation via protein kinaseB (AKT)/mammalian target of rapamycin (mTOR), which directly induces phosphorylation, is a prominent upstream regulator of MAF1 that directly induces phosphorylation of MAF1 and controls its localization and transcriptional activity. Phosphatase and tensin homolog (*PTEN*) is a major tumor suppressor and inhibitor of mTOR signaling that is activated by MAF1 [20]. Palian et al. [21] demonstrated that *MAF1* is a critical downstream target of *PTEN* that drives both its tumor suppressor and metabolic functions via the MAF1 protein without the involvement of MAF1 mRNA, suggesting that the abundance of MAF1 protein is tightly controlled in cancer cells. MAF1 expression is diminished by the loss of PTEN. Li et al. [22] showed that the PI3K/PTEN/AKT/mTOR pathway is a central controller of cell growth and a key driver of human cancer. Sun et al. [23] showed that a solid lipid nano-formulation of astaxanthin inhibits gland carcinogenesis via the mTOR/MAF1/PTEN pathway. Liu et al. [24] showed that PTEN lipid phosphatase activity enhances dengue virus production via AKT/FOXO1/MAF1 signaling. Palian et al. [21] also emphasized that MAF1/PTEN activity can be mediated by PI3K/AKT/FOXO1 signaling, suggesting a new pathway for regulating Pol III-dependent genes. However, while various mechanisms involving the AKT/FOXO1/MAF1 regulatory axis have been studied in cancer, they have not been studied in sepsis.

In our present study, we hypothesized that the Pol III-RIG-I-induced BBB injuries that occur in SAE may be involved with the loss of *MAF1* expression. We then further explored that hypothesis both in vitro and in vivo.

## Subjects and methods

### Experimental animals

A total of 40 specific pathogen-free female Sprague–Dawley (SD) rats (age, 8 weeks; weight range, 200–250 g) were obtained from the Experimental Animal Center of Sun Yat-sen University (Guangzhou, China). The rats were randomly assigned to five groups, which included a sham-operation group (sham, *n* = 8), a sepsis model group (model, *n* = 8), a sham group injected with a negative control lentivirus (1×10^8^ PFU/mL, 100 μL, sham + Lv-shCtrl, *n* = 8) via the carotid artery, a sepsis model group injected with a negative control (1×10^8^ PFU/mL, 100 μL, model + Lv-shCtrl, *n* = 8), and a sepsis model group injected with Lv-shCUL2 (1×10^8^ PFU/mL, 100 μL, model + Lv-shCUL2, *n* = 8). All lentiviruses were purchased from GenePharma (Suzhou, China). The rats were housed in separate cages in a facility maintained at room temperature and with a 12 h/12 h light/dark cycle. Food and water were available ad libitum. The experiments were approved by Guangdong Provincial People’s Hospital (KY-Z-2022-2284-03).

The sepsis model was constructed using the cecum ligation and puncture (CLP) method, as previously described [25]. Briefly, the rats were anesthetized by intraperitoneal injection of pentobarbital sodium (30 mg/kg), fixed in position, and disinfected. Next, a 1.5 cm incision was made along the midline of the abdomen and the cecum was exposed. In the model group, the cecum was ligated with no. 4 surgical sutures and pierced with a 3-mm-wide cone at approximately 0.8–1 cm from the distal end of the ligation. At the end of the operation, the cecum was returned to the abdominal cavity and the abdominal incision was closed layer by layer. The sham group of rats only underwent abdominal incision and suturing, without CLP. All animals were euthanized through intraperitoneal injection with an overdose of pentobarbital sodium 24 h post-surgery.

### RNA sequencing

The total RNA extracted from rat brains was tested for RNA integrity using Agilent Bioanalyzer 2100 (Agilent Technologies, Santa Clara, CA, USA). The construction of the sequencing sample library was then completed through the following steps: poly-A enrichment, mRNA fragmentation, first-strand cDNA synthesis, magnetic bead purification of the first strand, second-strand cDNA synthesis, strand end repair, 3′-end poly-A addition, addition of ligation adapters, purification, and library amplification. Subsequently, the sequencing was completed on an Illumina NovaSeq 6000 sequencer (Illumina, San Diego, CA, USA) according to the operating instructions. The raw image data files obtained from high-throughput sequencing were converted into raw reads through CASAVA base recognition. We used Stringtie software (Johns Hopkins University Center) to count the fragments within each gene segment and then normalized them using the trimmed mean of *M* values algorithm and converted them into gene expression levels using fragments per kilobase of transcript per million. Finally, differential analysis of gene expression (*P* < 0.05, fold change ≥ 2 or ≤ 0.5) between groups was performed using the edgeR software package.

### Cell culture

Rat brain microvascular endothelial cells (rBMECs, cat. no. CP-R108), rat brain astrocytes (rAstrocytes, cat. no. CP-R137), and rat cortex neuron cells (cat. no. CP-R105) were purchased from Procell Life Science & Technology Co., Ltd. (Wuhan, China) and used for in vitro experiments. The rBMECs and rAstrocytes were cultured in a 5% CO_2_ atmosphere for 24 h at 37 °C in a Dulbecco’s modified Eagle medium (DMEM) containing 10% fetal bovine serum and 1% penicillin/streptomycin, while the neuron cells were maintained in DMEM medium (Neurobasal + B27 + glutamine) containing 10% fetal bovine serum. To simulate the microenvironment of cells during sepsis, cells were stimulated with 10% serum harvested from the sham or model rats.

### Vector construction and transfection

The truncated leptin promoter and inhibitor fragments were amplified, inserted into a pGL3-Basic vector (Promega, Madison, WI, USA) and ligated by T4 DNA ligase (TaKaRa, Tokyo, Japan). When the cultured rBMECs and rAstrocytes reached 80–90% confluence, they were transfected with the CUL2, PTEN, or MAF1 overexpression vectors or the inhibitor plasmid vectors (shCUL2, 5′-UCUUAUACUGUUCAACAUGAA-3′; siFOXO1, 5′-AGCAAAUUUACUGUUGUUGUC-3′; GenePharma) by using a Lipofectamine 2000 kit (Thermo Fisher Scientific Inc., Waltham, MA, USA). Following transfection, the cells were treated with 10% serum obtained from rats in the sham and model groups. In this present study, rBMECs and rAstrocytes were also treated with Akt1 and Akt2-IN-1 inhibitors (10 μM, MedChemExpress, Shanghai, China) and protein synthesis inhibitors [cycloheximide (CHX), 1.0 μM, MedChemExpress].

### Hematoxylin and eosin (H&E) and immunohistochemical (IHC) staining

Tissue samples from rats in the model and sham groups were precooled in heparinized physiological saline and 4% paraformaldehyde, paraffin-embedded, and then cut into 4-μm-thick coronal sections. Next, H&E staining was performed by using an H&E staining kit (Solarbio, Beijing, China) according to the manufacturer’s instructions.

As for IHC staining, sections of the brain, myocardium, liver, kidney, and lung tissue were formalin-fixed and paraffin-embedded, deparaffinized, and gradually rehydrated. The sections were then incubated with primary antibodies against MAF1 (1:100, sc-515614, Santa Cruz Biotechnology, Dallas, TX, USA), CUL2 (1:50, sc-166506, Santa Cruz Biotechnology), and PTEN (1:100, BM4114, Boster, Wuhan, China) at 4 °C for 12 h. Next, the sections were incubated with a horseradish peroxidase (HRP)-labeled goat anti-rabbit IgG secondary antibody (1:20,000, BA1054, Boster) at room temperature for 40 min, and the numbers of positive cells were recorded.

### Evans blue dye (EBD) extravasation

Destruction of the BBB can cause increased permeability of the capillaries in brain tissue. Two hours before sacrifice, each rat received an intravenous injection of 2% EBD. One day later, each rat was anesthetized with 1% tombabic sodium (40 mg/kg), after which, the chest was opened, blood was collected, and the rat was perfused with physiological saline and heparin sodium (20 U/mL). Finally, the brain tissues were obtained and photographed. The EBD absorbance at 620 nm was measured by EBD fluorescence.

### Real-time quantitative PCR (qPCR)

The levels of *MAF1*, *CUL2*, *IFNβ*, *PTEN*, and *FOXO1* expression were evaluated both in vivo and in vitro. The total RNA in tissues and cells was extracted using TRIzol reagent (Invitrogen, Carlsbad, CA, USA). The primers used for qPCR were designed and synthesized by Sangon (Shanghai, China). The qPCR reaction was performed by using a HiScript II One Step qRT–PCR SYBR Green Kit (cat. no. Q221-01; Vazyme Biotech Co. Ltd., Nanjing, China) on an ABI 7900 PCR system (Foster City, CA, USA). Relative levels of gene expression were calculated using the 2^–ΔΔCt^ method. *GAPDH* served as an internal reference gene. All experiments were performed in triplicate.

### Western blotting

The levels of MAF1, CUL2, CD31, GFAP, caspase-3, RIG-I, IRF-3, p62, LC3B, AKT, p-AKT, p-mTOR, and PTEN protein expression were evaluated by western blotting. The protein concentrations in cell and tissue extracts were determined using a Pierce BCA Protein Assay kit (no. 23227; Thermo Scientific, Waltham, MA, USA).

An aliquot of protein from each sample was separated by sodium dodecyl sulfate–polyacrylamide gel electrophoresis (SDS–PAGE) performed at 120 V. The separated protein bands were transferred onto polyvinylidene fluoride (PVDF) membranes, which were subsequently washed with Tween-20: TBS at 1:1000 for 5 min and then blocked with 5% powdered skimmed milk at 4 ℃ overnight. Next, the membranes were incubated for 1 h with primary antibodies against MAF1, CUL2, CD31 (1:2000, ab222781, Abcam, Cambridge, MA, USA), GFAP (1:1000, ab279291), caspase-3 (1:2000, ab184787), RIG-I (1:1000, ab180675), IRF-3 (1:1000, ab238521), p62 (1:2000, PB0458), LC3B (1:1000, PA01524), AKT (1:500, ab8805), p-AKT (1:500, ab38449), p-mTOR (1:2000, ab109268), PTEN, and GAPDH (1:5000; ab8245) and subsequently incubated with a secondary antibody [horseradish peroxidase (HRP)-labeled goat anti-rabbit IgG]. The PVDF membranes were then incubated with electrogenerated chemiluminescence (ECL) solution (ECL808-25, Biomiga, San Diego, CA, USA) for 1 min and exposed to X-ray film. The net density value of each band was calculated using Image-Pro Plus 6.0 software, with GAPDH serving as an internal reference band. All experiments were repeated three times.

### Flow cytometric analysis

Flow cytometric analyses were performed to evaluate the levels of CD31 and GFAP in rBMECs and rAstrocytes. The cells were washed with precooled phosphate buffered saline (PBS), fixed with precooled 70% ethanol, and then stored at 4 ℃ overnight. Next, the cells were treated with propidium iodide [(PI) 25 μg/mL; Keygen, Nanjing, China] and RNase A (200 μg/mL) in the dark, and the cell cycle was analyzed using a flow cytometer (BD Biosciences, San Jose, CA, USA).

### Transendothelial electrical resistance (TEER)

TEER was considered a marker for evaluating blood–brain barrier permeability. The rBMECs and rAstrocytes were digested and counted. About 1 × 10^5^ rBMECs and 1 × 10^5^ rAstrocytes were seeded into a Transwell chamber (upper) and inserted into a 24-well plate at 37 ℃, 5% CO_2_. In the lower chamber, about 1 × 10^4^ rat cortex neuron cells were added and co-cultured with the cells in the upper chamber. A Millicell ERS-2 Voltohmmeter (Merck Millipore, Billerica, MA, USA) was employed to measure the cellular monolayers once every 2 days. The baseline reading was recorded, and the TEER was calculated.

### Transmission electron microscope (TEM)

TEM was used to assess cell autophagy. The rBMECs and rAstrocytes were sequentially fixed in 2.5% glutaraldehyde and 2% osmium tetroxide buffer, after which, they were dehydrated in a graded ethanol series and embedded in Epon. A JEM-100CX-II TEM transmission electron microscope (Joel, Tokyo, Japan) was used to observe the cells.

### Electrophoretic mobility shift assay (EMSA)

Complementary oligonucleotide probes targeting the PTEN promoter were synthesized. Additionally, 5′-biotinylated, competitive probes without 5′-biotinylation, and mutant competitive probes were also synthesized. Next, the nuclear proteins were extracted and incubated with the probes. The electrophoretic mobility shift assay (EMSA) was performed using a LightShift Chemiluminescent EMSA kit (Pierce, Rockford, IL, USA) according to the manufacturer’s instructions.

### Statistical analyses

All statistical and image analyses were performed using GraphPad Prism 9 software (San Diego, CA, USA). An unpaired student’s *t*-test and one-way analysis of variance (ANOVA) were used to evaluate differences, depending on the number of groups. Data are expressed as a mean value ± standard deviation (SD). *P* < 0.05 was considered to be statistically significant.

## Results

### *MAF1* and *CUL2* were dysregulated in the brains of sepsis models

**Fig. 1:**
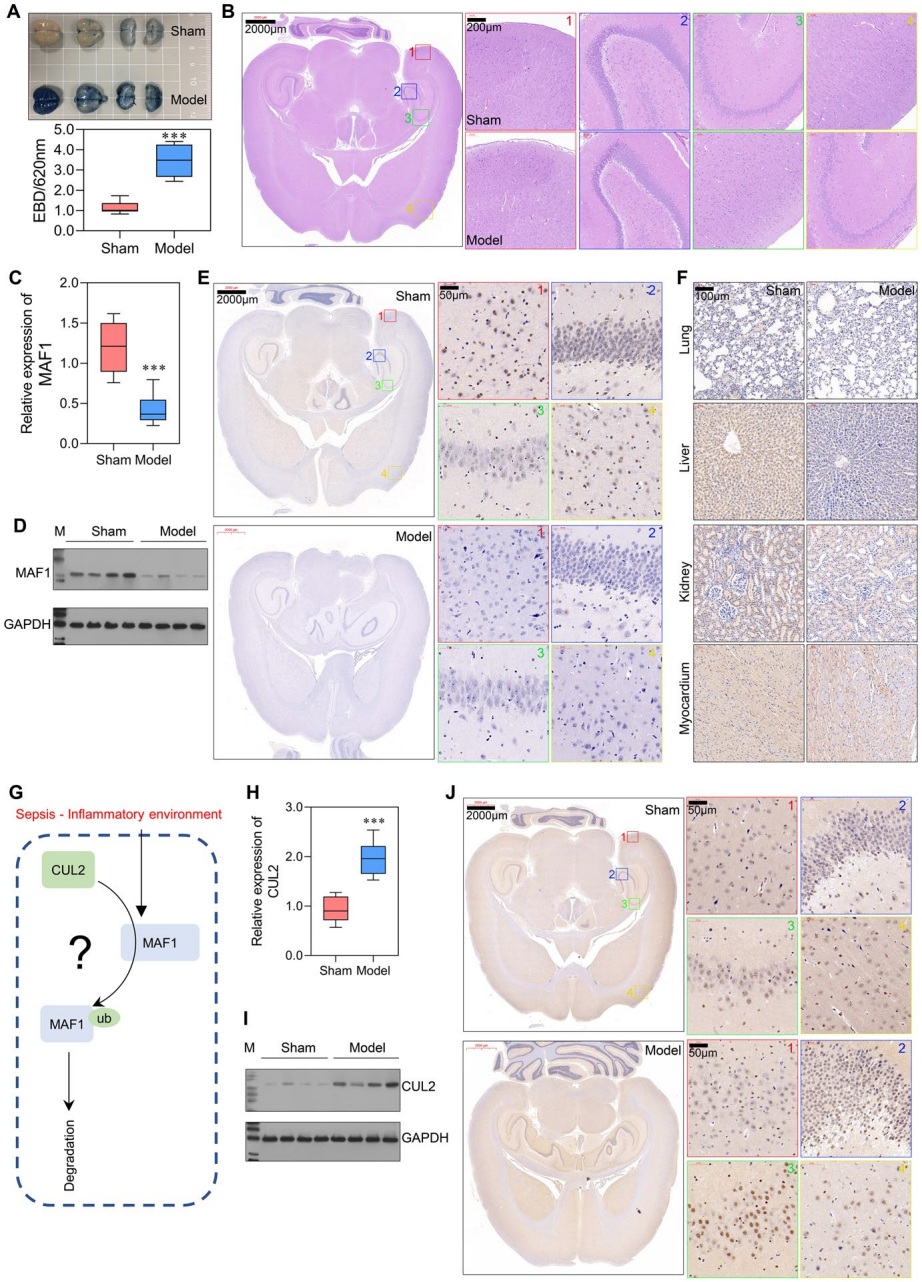
*MAF1* and *CUL2* were dysregulated in rats with sepsis. Sepsis rat models were constructed by cecal ligation and puncture, and brain tissue was isolated after 24 h. **A** EBD extravasation and absorbance at 620 nm in brain tissues from the sham and model groups. **B** H&E staining of brain tissue. **C**, **D** MAF1 expression in the whole brain evaluated by qPCR and western blotting. **E** MAF1 expression in four regions is shown by IHC staining. **F** IHC analysis of MAF1 expression in lung, liver, kidney, and myocardium tissues. **G** The hypothesis that *CUL2* promotes the ubiquitination degradation of MAF1 in septic-induced inflammatory environments. **H** *CUL2* expression as evaluated by qPCR and western blotting. **I** CUL2 expression in four regions is shown by IHC staining. ***P < 0.001

In the present study, we first constructed the in vivo models. As shown in Fig. 1A, rats in the model group displayed more intense EBD staining than rats in the sham group, indicating high permeability and severe damage to the BBB in the model group. In addition, H&E staining of brain tissues showed decreased numbers of neuronal cells in the cortical layer and a shrunken and disordered arrangement of the nucleus in the model group (Fig. 1B). Through transcriptome high-throughput sequencing, we identified a large number of differentially expressed genes, which are involved in multiple signaling pathways (Fig. S1A–S1E). In the top ten genes with differential expression, we observed that the expression level of MAF1 was significantly reduced in sepsis rats when compared with control rats. Results of qPCR and western blotting showed that *MAF1* was expressed at significantly lower levels in the model group when compared with the sham group (Fig. 1C, D). The levels of MAF1 expression were also evaluated by IHC and immunofluorescence assay in four different areas. All four areas showed decreased MAF1 expression in the model tissues (Fig. 1E, S1F). Furthermore, MAF1 expression was measured in the myocardium, liver, kidney, and lung tissues. As shown in Fig. 1F, MAF1 was expressed at significantly lower levels in the model group than in the sham group in myocardial, liver, and renal tissues. However, there was no difference in the levels of MAF1 expression in the lung tissues of the two groups.

To investigate whether the expression level of *MAF1* in the blood–brain barrier was influenced by the inflammatory environment induced by sepsis, the rBMECs (90.9% CD31^+^) and rAstrocytes (96.8% GFAP^+^) were isolated and identified for an in vitro experiment (Fig. S2A). Results of western blot studies were consistent with those shown by flow cytometry (Fig. S2B). Next, the rBMECs and rAstrocytes were treated with 10% serum isolated from the sham and model rats. The results of the CCK-8 assay showed that cellular activity gradually decreased with increasing treatment time in the model-S group, and there were especially significant differences between the sham-S and model-S groups after 24 h of treatment (Fig. S2C). In addition, qPCR results showed that *MAF1* expression was downregulated in the model-S group after graded treatment and there was a significant difference between the sham-S and model-S groups at 24 h of treatment (Fig. S2D). Of note, the western blot results for the protein level of MAF1 (Fig. S2E, S2F) were significantly reduced after 6 h, which is different from the results obtained by qPCR. These results suggest that the transcriptional regulation and post-translational modification of MAF1 may both be influenced by serum stimulation. Furthermore, we established a co-cultivation system and found that the serum from rats with sepsis altered the permeability and TEER value of membranes composed of rBMECs and rAstrocytes (Fig. S2G, S2H). There were significant differences in IFNβ concentration and neuronal activity in the lower chamber (Fig. S2J, S2K). Wang et al. [13] reported that E3 ubiquitin ligase *CUL2* critically regulates MAF1 ubiquitination and controls its stability. Hence, we suggested a hypothesis that the ubiquitin–proteasome degradation of MAF1 might be changed, and *CUL2* might be an essential regulator of that (Fig. 1G). The results showed that the levels of CUL2 in brain tissues from the model group were significantly higher than in brain tissues from the sham group (Fig. 1H–J). In summary, this suggests that *MAF1* and *CUL2* expression were dysregulated during BBB injury in sepsis.

### Knockdown of *CUL2* stimulated *MAF1* expression and attenuated the RIG-I/IRF3 signaling pathway

**Fig. 2:**
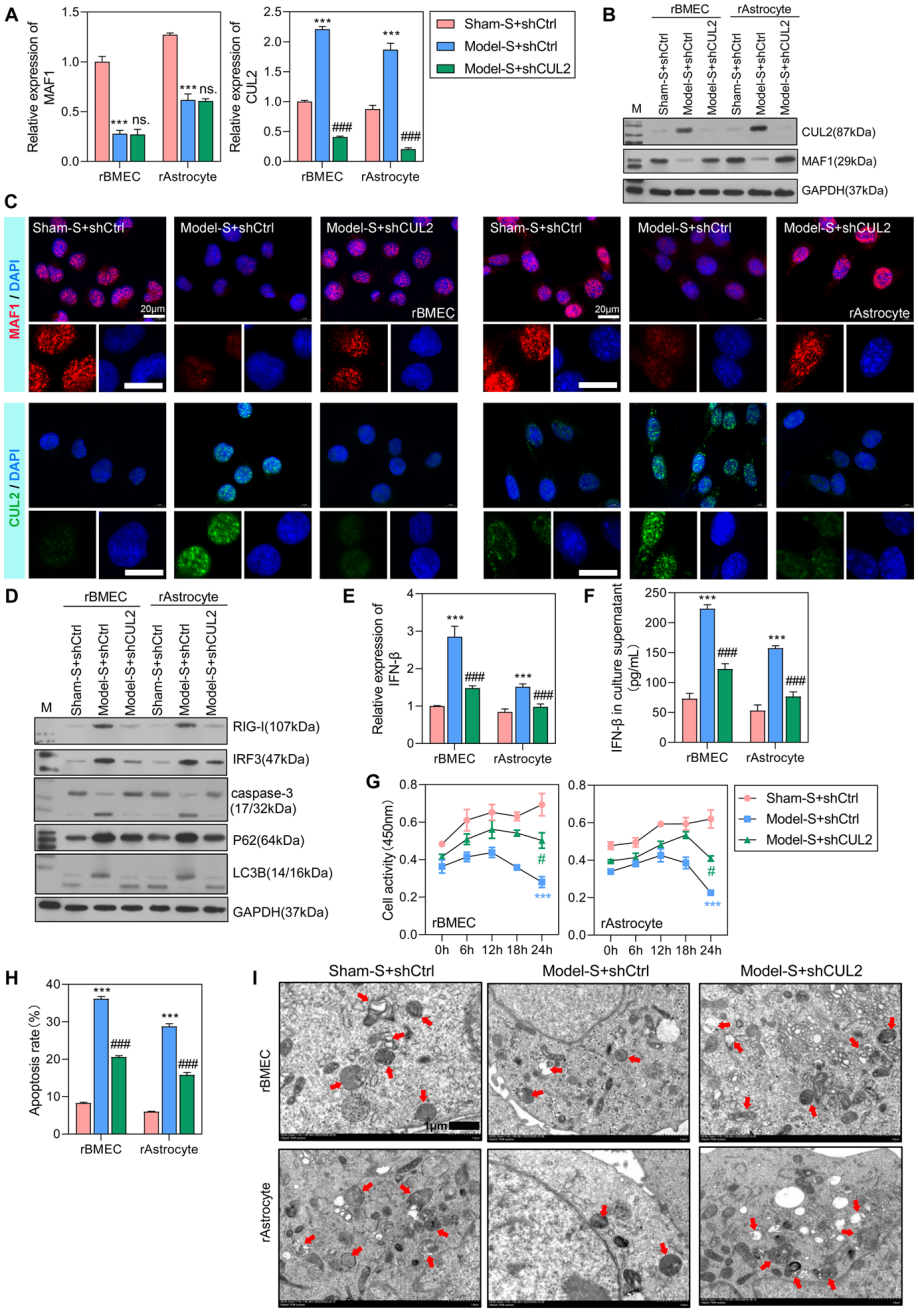
Knockdown of *CUL2* stimulated *MAF1* expression, attenuated cell apoptosis, and recovered normal autophagy. The rBMECs and rAstrocytes were treated with 10% serum harvested from the sham (sham-S) or model rats (model-S). **A**, **B** qPCR and western blotting studies of *MAF1* and *CUL2* expression in the sham-S + shCtrl, model-S + shCtrl, and model-S + shCUL2 groups. **C** MAF1 and CUL2 expression was measured by IF. **D** Western blot analyses of RIG-I, IRF3, caspase-3, p62, and LC3B protein expression. **E**, **F** *IFNβ* expression levels were detected by qPCR and enzyme-linked immunosorbent assay (ELISA). **G** CCK-8 analyses of rBMEC and rAstrocyte cellular activity after transfection for 0, 6, 12, 18, and 24 h. **H** Flow cytometry was used to detect the apoptosis of transfected cells. **I** Transmission electron microscopy was used to detect autophagy in rBMECs and rAstrocytes ***P < 0.001 model-S + shCtrl versus sham-S + shCtrl. #*P* < 0.05, ###*P* < 0.001 model-S + shCUL2 versus sham-S + shCtrl

To further investigate the regulatory effect of *CUL2* on *MAF1*, in vitro transfections were performed. Compared with the level of *MAF1* expression in the sham-S + shCtrl group, *MAF1* expression was significantly decreased in model-S + shCtrl and model-S + shCUL2 groups (Fig. 2A). Moreover, *CUL2* expression was dramatically increased in the model-S + shCtrl group and decreased in the model-S + shCUL2 group when compared with the sham-S + shCtrl group. Next, CUL2 protein expression was consistent with the results obtained from the qPCR assay, while MAF1 protein expression was increased in the model-S + shCUL2 group (Fig. 2B, C), which was different from the results of qPCR assays. These results indicate that *MAF1* expression in the model-S group was increased after knockdown of *CUL2*.

We also examined trends in the expression of downstream proteins, RIG-I and IRF3, and found that the expression levels of both proteins were increased in the model-S + shCtrl group and decreased in the model-S + shCUL2 group when compared with the sham-S + shCtrl group (Fig. 2D). These changes in expression were the opposite of those shown by the MAF1 protein. A downstream biomarker of the RIG-I/IRF3 signaling pathway (IFNβ) was also measured. The levels of IFNβ expression were significantly increased in the model-S + shCtrl group and decreased in the model-S + shCUL2 group when compared with the sham-S + shCtrl group (Fig. 2E). The levels of IFNβ in culture supernatants were consistent with the *IFNβ* expression levels (Fig. 2F). The apoptosis-related protein caspase-3 was only slightly activated in the model-S + shCUL2 group and highly activated in the model-S + shCtrl group, indicating an attenuated apoptosis function after the knockdown of *CUL2* (Fig. 2D). Moreover, we analyzed the effect of CUL2 on cell autophagy. The p62 protein combines with the LC3B protein to form a complex, which eventually becomes degraded. Our study found that expression of the autophagy substrate p62 protein was decreased, while expression of the autophagy-related protein LC3B-II was increased in the model-S + shCUL2 group (Fig. 2D), indicating an average level of autophagy. A cell activity analysis showed that the cellular activities of the rBMECs and rAstrocytes in the model-S + shCtrl and model-S + shCUL2 groups were significantly suppressed when compared with cellular activity in the sham-S + shCtrl group at 24 h of treatment (Fig. 2G). The apoptosis rates detected by flow cytometry were consistent with the western blot analyses (Fig. 2H). As shown in Fig. 2I, the autophagy levels were decreased in the model-S + shCtrl group but were at an average level in the model-S + shCUL2 group. Therefore, we conclude that the knockdown of *CUL2* stimulated *MAF1* expression attenuated the RIG-I/IRF3 signaling pathway, enhanced cell apoptosis, and recovered normal autophagy.

### *CUL2* overexpression promoted the degradation of MAF1

**Fig. 3:**
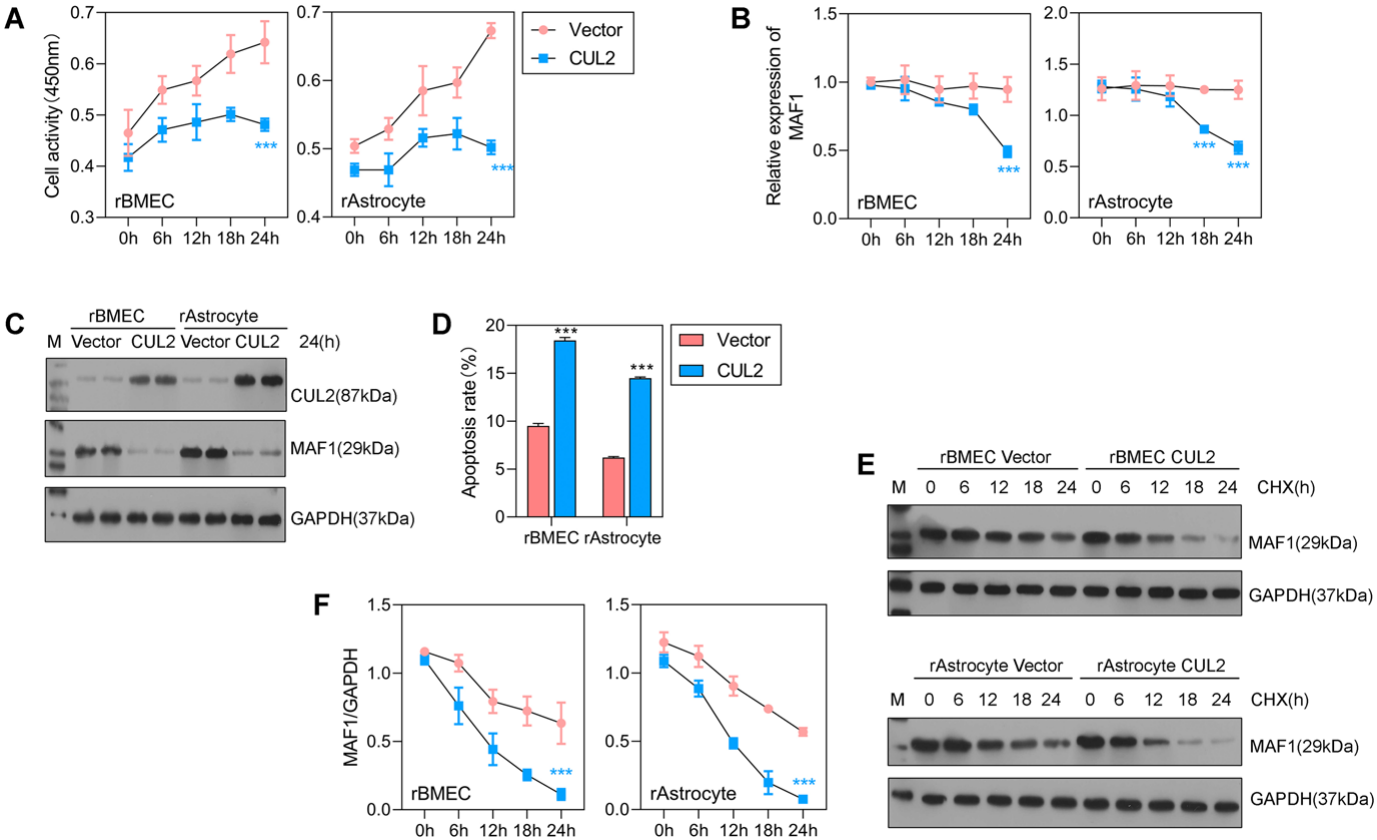
*CUL2* overexpression promoted the degradation of MAF1. **A** CCK-8 analysis of rBMECs and rAstrocytes transfected with the *CUL2* overexpression vector or empty vector. **B**, **C** qPCR and western blot analyses were performed to detect the expression levels of *MAF1* and *CUL2* in cells transfected with the empty vector or *CUL2* overexpression vector. **D** Flow cytometry was used to detect the apoptosis of cells transfected with the empty vector or *CUL2* overexpression vector. **E**, **F** The levels of *MAF1* in cells cotransfected with the *CUL2* overexpression vector and *MAF1* overexpression vector and treated with cycloheximide (CHX) (10 μg/mL). ***P < 0.001

Subsequently, we overexpressed *CUL2* in rBMECs and rAstrocytes and then assessed the levels of cellular activity and apoptosis in the transfected cells. Our results showed that overexpression of *CUL2* significantly inhibited cellular activity after 24 h of treatment (Fig. 3A). The expression of *MAF1* mRNA was significantly decreased in the rBMECs + CUL2 and rAstrocytes + CUL2 groups when compared with the rBMECs + vector and rAstrocytes + vector groups (Fig. 3B). Moreover, MAF1 protein expression was also significantly inhibited by overexpression of *CUL2* (Fig. 3C). Flow cytometry results demonstrated that overexpression of *CUC2* increased the rate of cell apoptosis (Fig. 3D). These results indicate that overexpression of *CUL2* inhibits *MAF1* expression, suppresses cell activity, and promotes cell apoptosis.

Next, we forced the overexpression of both *CUL2* and *MAF1* in rBMECs and rAstrocytes. At 1 day after transfection, the cells were treated with a protein synthesis inhibitor [cycloheximide (CHX)] and then harvested at 0, 6, 12, 18, and 24 h. We found that the levels of MAF1 protein and mRNA expression were both significantly decreased in the rBMECs + CUL2 + MAF1 + CHX group and rAstrocytes + CUL2 + MAF1 + CHX group when compared with the rBMECs + MAF1 + CHX and rAstrocytes + MAF1 + CHX groups after 24 h of CHX treatment (Fig. 3E, F). After suppression of mRNA translation by CHX, MAF1 expression declined at a more rapid rate in the *CUL2* overexpression group than in the vector group, indicating that *CUL2* regulates the stability and promotes the degradation of MAF1. In addition, we found that the expression of *CUL2* and *MAF1* was both associated with the permeability and TEER value of membranes composed of rBMECs and rAstrocytes (Fig. S3A–S3H), which indicates that abnormal expression of *CUL2* may promote blood–brain barrier dysfunction.

### Knockdown of *CUL2* reduced BBB damage in SAE rats

**Fig. 4:**
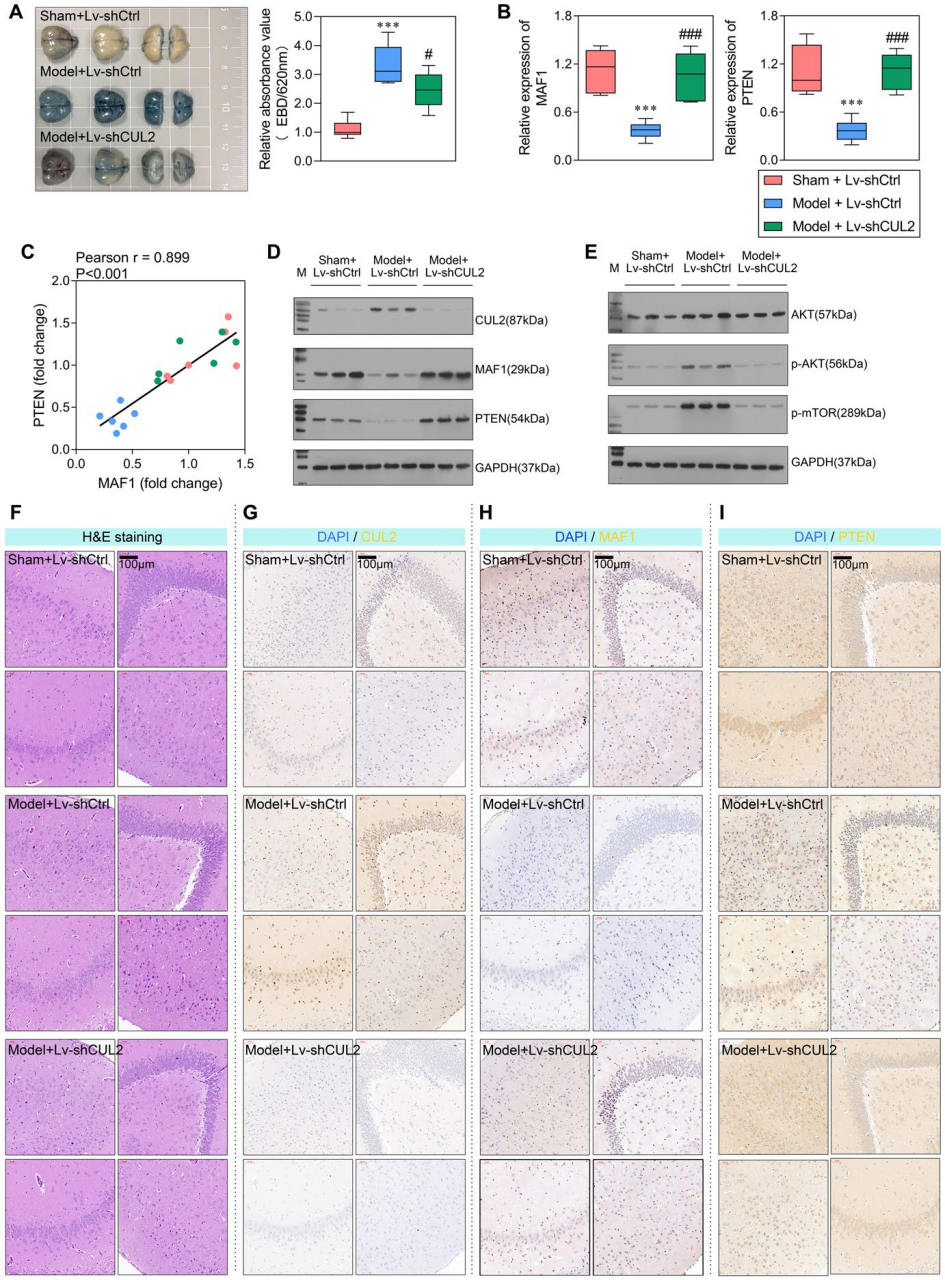
Knockdown of *CUL2* increased *MAF1* and *PTEN* expression and relieved cellular damage. **A** EBD extravasation and absorbance at 620 nm in brain tissues from the sham + Lv-shCtrl, model + Lv-shCtrl, and model + Lv-shCUL2 groups. **B** The levels of *MAF1* and *PTEN* expression were measured by qPCR. **C** A Pearson correlation analysis was performed on the expression levels of *MAF1* and *PTEN*. **D**, **E** Western blot analyses evaluating the levels of CUL2, MAF1, PTEN, AKT, p-AKT, and p-mTOR protein expression. **F** H&E staining showing the brain tissue morphology. **G**–**I** IHC analyses of CUL2, MAF1, and PTEN expression in brain tissues. ***P < 0.001 model + Lv-shCtrl versus sham + Lv-shCtrl. #*P* < 0.05, ###*P* < 0.001, model + Lv-shCUL2 versus model + Lv-shCtrl

As shown in Fig. 4A, brain tissues from the shCUL2 group showed less intense EBD staining than brain tissues from the shCtrl group. The relative absorbance value at 620 nm was significantly increased in the model + Lv-shCtrl group when compared with the sham + Lv-shCtrl group, and considerably decreased in the model + Lv-shCUL2 group when compared with the model + Lv-shCtrl group (Fig. 4A), indicating that knockdown of CUL2 resulted in less tissue permeability and relieved damage to the BBB. As a transcription factor of phosphatase and tensin homolog (PTEN), the levels of MAF1 and PTEN were assessed in vivo. The results showed that both levels were significantly increased by treatment with shCUL2 (Fig. 4B). Moreover, their expression levels showed a positive correlation (Pearson *r* = 0.899; *P* < 0.001; Fig. 4C). At the protein level, both expression trends were consistent with their mRNA levels (Fig. 4D). An IHC analysis revealed that the expression levels of CUL2, MAF1, and PTEN in brain tissues were consistent with their respective western blotting results (Fig. 4G–I).

We hypothesized that PTEN-AKT-mTOR might play an essential role in influencing *MAF1* function. Therefore, we measured the expression levels of AKT and mTOR in vivo and found that their expression levels were opposite to that of PTEN (Fig. 4E). The AKT/mTOR signaling pathway was attenuated by shCUL2. Therefore, PTEN might function by affecting mTOR and AKT phosphorylation. H&E revealed damage to brain tissues in the model + Lv-shCtrl group, but the damage could be rearranged by shCUL2 (Fig. 4F). Knockdown of *CUL2* increased MAF1 and PTEN expression, decreased AKT/mTOR signaling pathway activity, and relieved damage to the BBB.

### Overexpression of *PTEN* decreased cell apoptosis and normalized autophagy in vivo

**Fig. 5:**
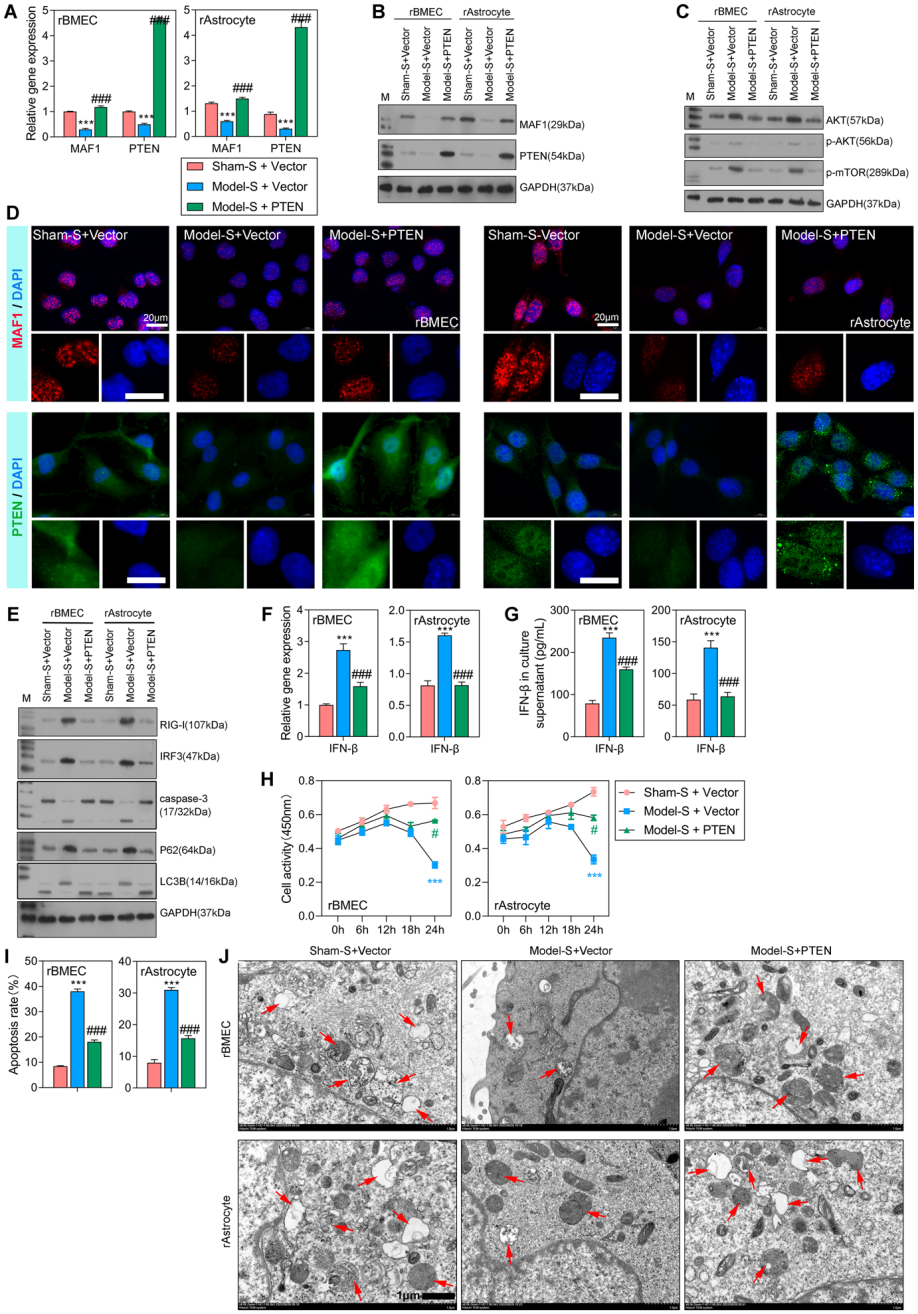
Overexpression of *PTEN* attenuated the RIG-I/IRF3 signal pathway and apoptosis and recovered normal autophagy. The rBMECs and rAstrocytes were treated with 10% serum harvested from the sham (sham-S) or model rats (model-S). **A**, **B** qPCR and western blot analyses of *MAF1* and *PTEN* expression in the sham-S + vector, model-S + vector, and model-S + PTEN groups. **C** Western blot analyses of AKT/mTOR signaling pathway. **D** MAF1 and CUL2 expression was measured by IF. **E** Western blot analyses of proteins RIG-I and IRF3, caspase-3, p62, and LC3B. **F**, **G** qPCR and flow cytometry were used to detect *IFNβ* expression levels. **H** CCK-8 analysis of rBMEC and rAstrocyte activity at 0, 6, 12, 18, and 24 h after transfection. **I** Flow cytometry analysis of apoptosis in transfected cells. **J** Transmission electron microscopy was used to detect autophagy in rBMECs and rAstrocytes. ***P < 0.001 model-S + vector versus sham-S + vector. #*P* < 0.05, ###*P* < 0.001 model-S + PTEN versus sham-S + vector

After confirming the relationship between *PTEN* and *MAF1*, we examined the mechanism by which these molecules affect injuries to the BBB. A *PTEN* overexpression vector was transfected into rBMECs and rAstrocytes that had been previously stimulated. The levels of *MAF1* and *PTEN* expression were significantly decreased in the model-S + vector group and increased in the model-S + PTEN group (*P* < 0.001, Fig. 5A, B, D). Overexpression of *PTEN* promoted *MAF1* expression, suggesting a positive regulatory effect of *MAF1* on *PTEN*. The levels of phosphorylated AKT and mTOR were increased in the model-S + vector group and decreased by overexpression of *PTEN* (Fig. 5C). The regulation of RIG-I and IRF3 was inhibited by overexpression of *PTEN* (Fig. 5E), as opposed to the effect of MAF1. The apoptosis-related protein caspase-3 was highly expressed in the model-S + PTEN group and expressed at low levels in the model-S + vector group, indicating a promotional effect of *PTEN* on apoptosis. The levels of autophagy-related proteins p62 and LC3B were significantly reduced in the model-S + PTEN group when compared with the model-S + vector group, suggesting that overexpression of *PTEN* resulted in a normal level of autophagy. When *MAF1* expression was increased by overexpression of *PTEN*, the levels of RIG-I/IRF3, apoptosis, and autophagy became well-regulated. *IFNβ* expression was significantly increased in the model-S + vector group and decreased in the model-S + PTEN group when compared with the sham-S + shCtrl group (Fig. 5F). The levels of IFNβ in cell culture supernatants were consistent with *IFNβ* expression levels (Fig. 5G). A cell activity analysis showed that the metabolic activity of rBMECs and rAstrocytes in the model-S + vector and model-S + PTEN groups was significantly suppressed when compared with those cell types in the sham-S + vector group at 24 h of treatment (Fig. 5H). The apoptosis rate detected by flow cytometry was consistent with results from the western blot analysis (Fig. 5I). As shown in Fig. 5J, the autophagy level was decreased in the model-S + vector group, but then recovered to an average level in the model-S + PTEN group. In summary, overexpression of *PTEN* inhibited IFNβ production, increased cell activity, decreased cell apoptosis, and normalized autophagy.

### The AKT/mTOR signaling pathway regulates *MAF1* transcription

**Fig. 6:**
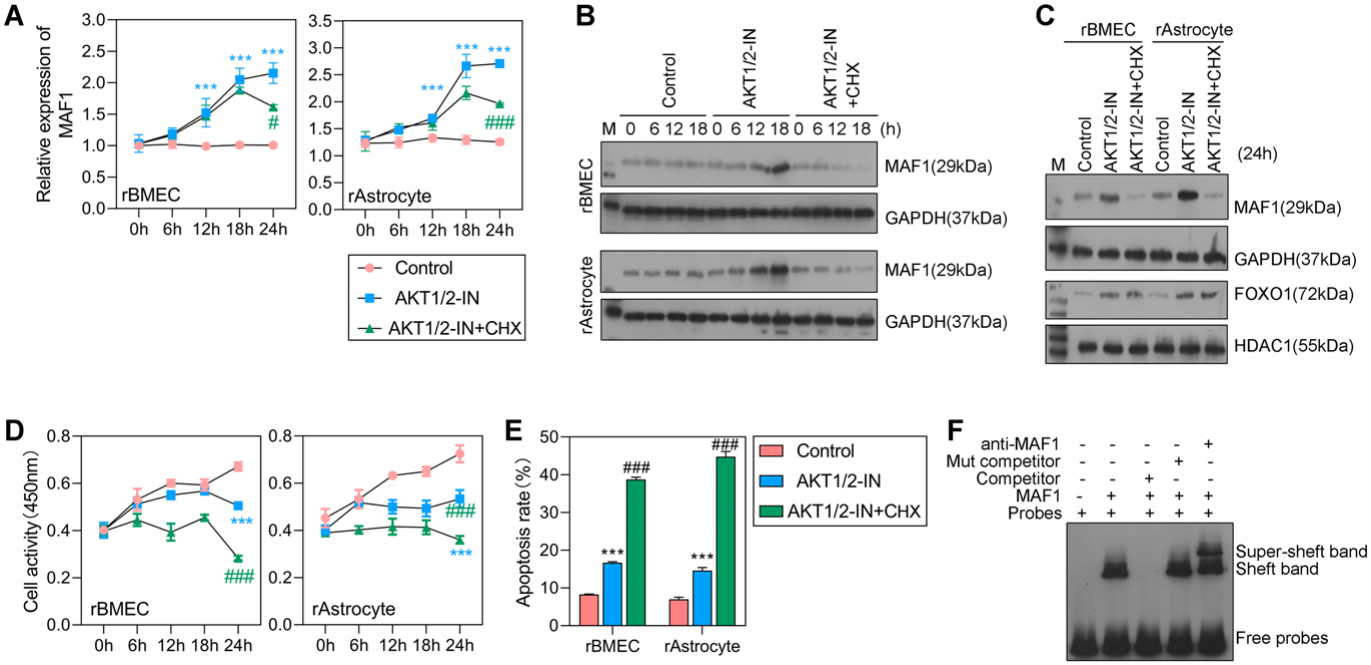
The AKT/mTOR signaling pathway inhibited cellular activity and promoted cell apoptosis. **A** Levels of *MAF1* expression were evaluated in the control, AKT1/2-IN, and AKT1/2-IN + CHX groups. **B**, **C** Western blot analyses were performed to assess the levels of MAF1 and FOXO1 protein expression in the control, AKT1/2-IN, and AKT1/2-IN + CHX groups. **D**, **E** The levels of cell activity and cell apoptosis in the control, AKT1/2-IN, and AKT1/2-IN + CHX groups. **F** An EMSA confirming the binding relationship between *MAF1* and the *PTEN* promoter. ***P < 0.001 AKT1/2-IN versus control. #*P* < 0.05, ###*P* < 0.001 AKT1/2-IN + CHX versus AKT1/2-IN

We assessed the effect of the AKT/mTOR pathway on SAE in vitro by co-culturing cells with inhibitors of Akt1 and Akt2-IN-1, as well as a protein synthesis inhibitor (clindamycin). Our data showed that *MAF1* mRNA expression was significantly increased in the AKT1/2-IN and AKT1/2-IN + CHX groups when compared with a control group (Fig. 6A). When the treatment lasted for 24 h, the levels of MAF1 expression began to decrease in the cells treated with clindamycin. These results indicated that *MAF1* mRNA expression was significantly promoted by the AKT kinase inhibitors, but was not rapidly affected by protein translation inhibitors. The expression levels of MAF1 and FOXO1 proteins gradually increased in the AKT1/2-IN group and progressively decreased with increasing treatment time in the AKT1/2-IN + CHX group (Fig. 6B, C), indicating that the AKT/mTOR signaling pathway regulates *MAF1* transcription. Next, we further investigated the role played by the AKT/mTOR signaling pathway in cell activity and apoptosis. We found that treatment with the AKT kinase inhibitor significantly inhibited cellular activity but accelerated cell apoptosis (Fig. 6D, E). An EMSA was performed to test the binding relationship between *MAF1* and the *PTEN* promoter. The results showed that the hypermigration test was also performed with anti-MAF1 antibody, whereas the unlabeled *PTEN* fragment was competitively inhibited (Fig. 6F). These results suggest that *MAF1* can regulate *PTEN* expression by directly targeting the *PTEN* promoter. After treatment with the protein translation inhibitor, the level of cell activity was further diminished, and cell apoptosis was further promoted. Taken together, these findings indicate that the AKT/mTOR signaling pathway regulates *MAF1* transcription, inhibits cellular activity, and promotes cell apoptosis.

### Interference with *FOXO1* inhibited *MAF1* expression

**Fig. 7:**
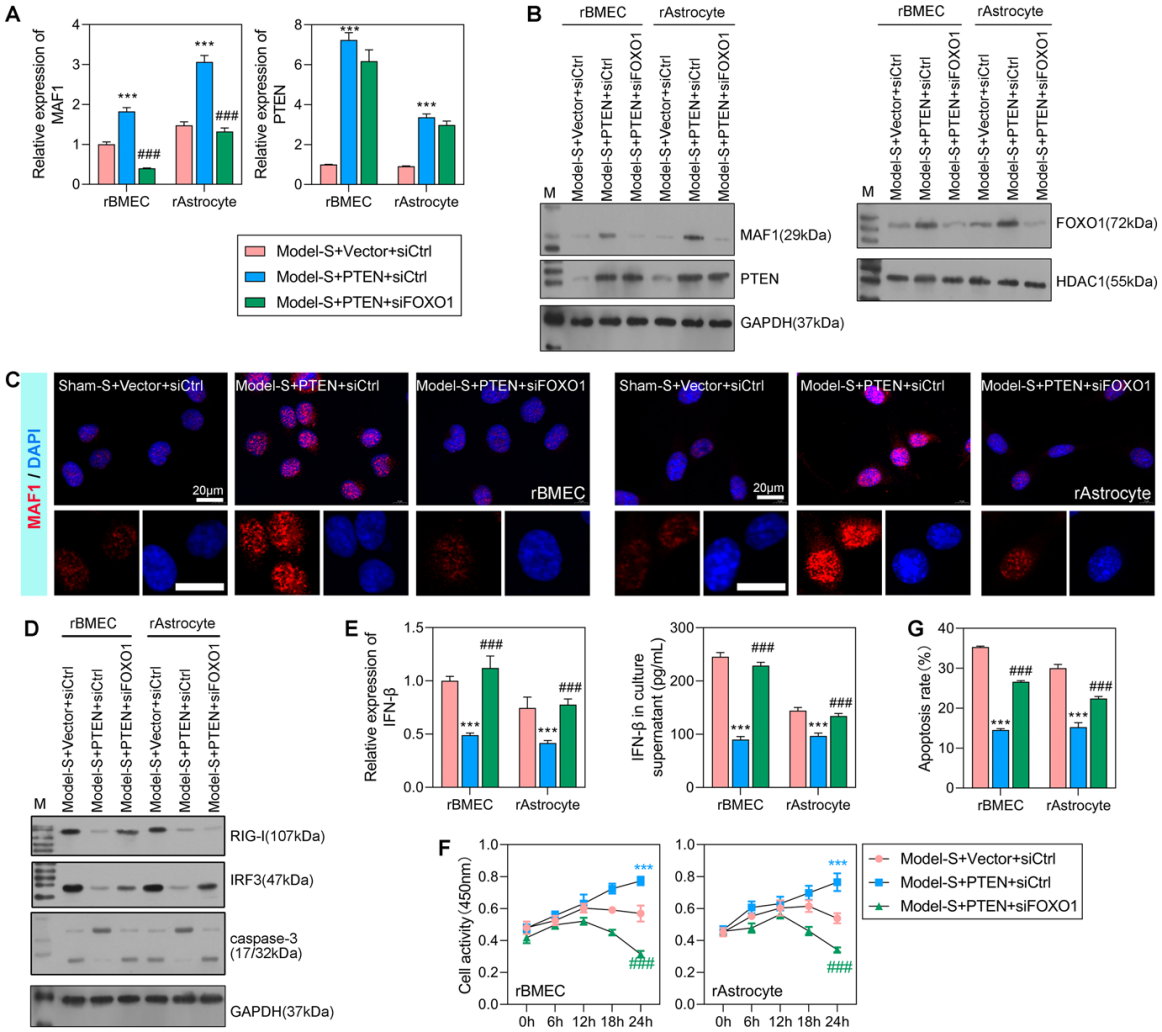
Interference with *FOXO1* inhibited cellular activity and enhanced cell apoptosis. The rBMECs and rAstrocytes were treated with 10% serum harvested from the sham (sham-S) or model rats (model-S). **A**, **B** qPCR and western blot analyses of *MAF1*, *PTEN*, and *FOXO1* expression in the model-S + vector + siCtrl, model-S + PTEN + siCtrl, and model-S + PTEN + siFOXO1 groups. **C** MAF1 expression was measured by IF. **D** Western blot analyses evaluating the protein expression levels of RIG-I and IRF3 and apoptosis-related protein caspase-3. **E** qPCR and flow cytometry were used to detect *IFNβ* expression levels. **F** CCK-8 analyses for detection of cellular activity in rBMECs and rAstrocytes after transfection for 0, 6, 12, 18, and 24 h. **G** Flow cytometry was used to detect the apoptosis of transfected cells. ***P < 0.001 model-S + PTEN + siCtrl versus model-S + vector + siCtrl. #*P* < 0.05, ###*P* < 0.001 model-S + PTEN + siFOXO1 versus model-S + PTEN + siCtrl

Because *FOXO1* might be an intermediate factor in the AKT pathway that regulates *MAF1* transcription, we further assessed the PTEN/AKT/FOXO1/MAF1 pathway by overexpressing *PTEN* and interfering with *FOXO1* expression in rBMECs and rAstrocytes that had been treated with serum isolated from sham and model rats. The levels of *MAF1* mRNA and protein expression were significantly increased in the model-S + PTEN + siCtrl group when compared with the model-S + vector + siCtrl group (Fig. 7A–C). In addition, overexpression of *PTEN* promoted the expression of *FOXO* (Fig. 7B**)**. After interfering with *FOXO1*, the expression levels of MAF1 in rBMECs and rAstrocytes were significantly decreased when compared with those in the model-S + PTEN + siCtrl group. Furthermore, the expression levels of PTEN in both the model-S + PTEN + siCtrl and model-S + PTEN + siFOXO1 groups were significantly increased when compared with those in the model-S + vector + siCtrl group.

Subsequently, we examined three proteins that function downstream of MAF1-RIG-I, IRF3, and caspase-3, respectively. As shown in Fig. 7D**,** overexpression of *PTEN* decreased the levels of RIG-I and IRF3 expression; however, those levels could be increased by treatment with siFOXO1. The apoptosis-related protein caspase-3 was expressed at low levels in the model-S + PTEN + siCtrl group but was highly expressed in the model-S + PTEN + siFOXO1 group, indicating the promotive effect of siFOXO1 on apoptosis. *IFNβ* expression was significantly decreased in the model-S + PTEN + siCtrl group and increased in the model-S + PTEN + siFOXO1 group when compared with the model-S + Vector + siCtrl group (Fig. 7E); these trends in regulation were opposite to those of *MAF1*. Additionally, we found that siFOXO1 significantly inhibited cellular activity and promoted cell apoptosis (Fig. 7F, G). In summary, siFOXO1 inhibited MAF1 expression, decreased activity of the RIG-I/IRF3 signaling pathway, inhibited cellular activity, and enhanced cell apoptosis. Moreover, the opposing effects of siFOXO1 and *PTEN* overexpression on downstream proteins, cellular activity, and cell apoptosis demonstrate that *FOXO1* was involved in the positive regulation of *PTEN* by *MAF1*.

### Overexpression of *MAF1* decreased cell apoptosis and normalized autophagy

**Fig. 8:**
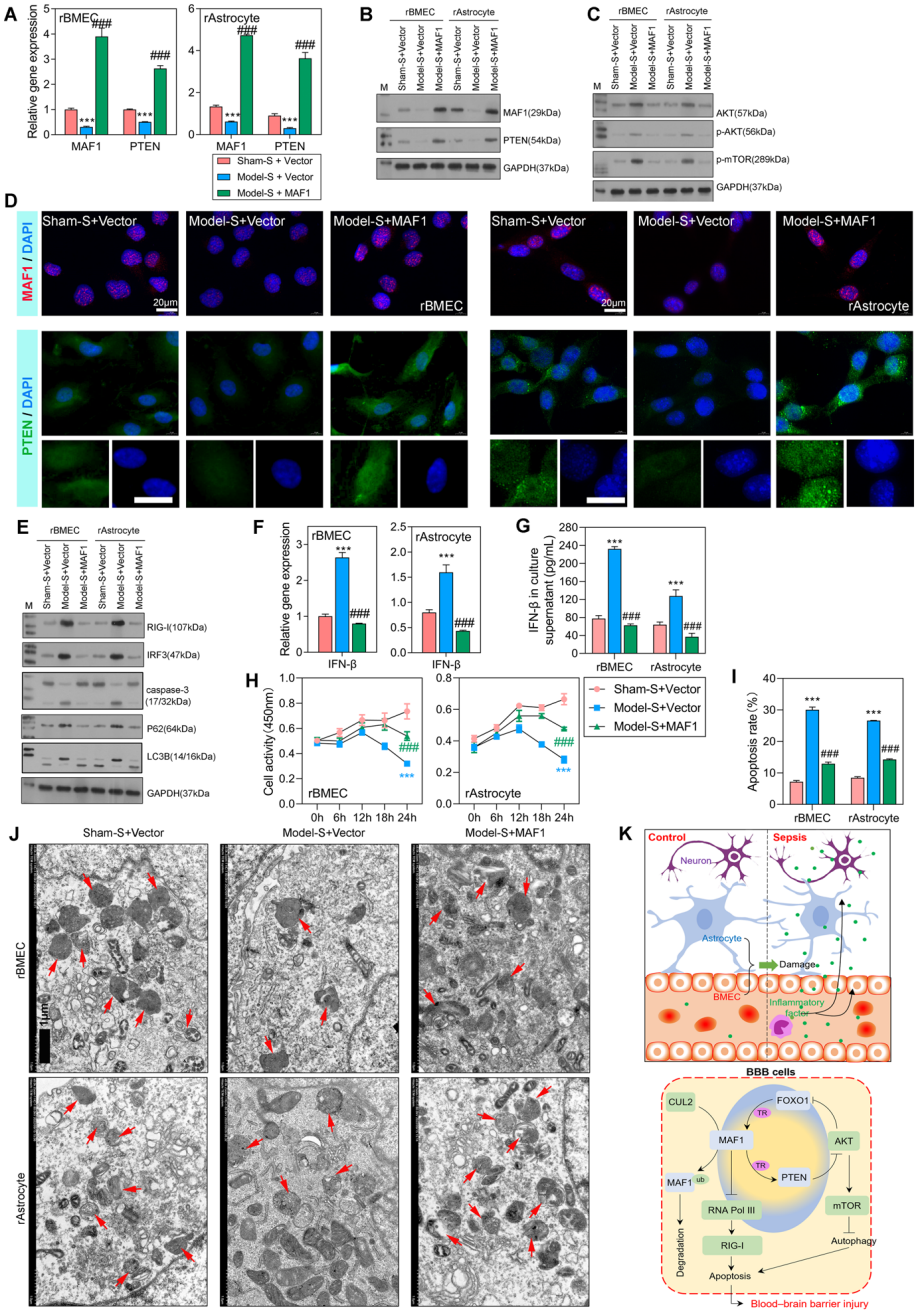
Overexpression of *MAF1* promoted *PTEN* expression, increased cellular activity, decreased cell apoptosis, and normalized autophagy. **A**, **B** qPCR and western blot analyses of *MAF1* and *PTEN* expression in the sham-S + vector, model-S + vector, and model-S + MAF1 groups. **C** Western blot analyses of AKT/mTOR signaling pathway proteins. **D** MAF1 and PTEM expression was measured by IF. **E** Western blot analyses of RIG-I and IRF3, the apoptosis-related protein caspase-3, and autophagy-related proteins p62 and LC3B. **F**, **G** qPCR and flow cytometry were used to detect *IFNβ* expression levels. **H** CCK-8 analyses for detection of cellular activity in rBMECs and rAstrocytes after transfection for 0, 6, 12, 18, and 24 h. **I** Flow cytometry was used to detect the apoptosis of transfected cells. **J** Transmission electron microscopy was used to detect autophagy in rBMECs and rAstrocytes. **K** Schematic diagram of the mechanism by which *MAF1* expression level affects BBB function in SAE. ***P < 0.001 model-S + vector versus sham-S + vector. #*P* < 0.05, ###*P* < 0.001 model-S + MAF1 versus sham-S + vector

Finally, the regulatory effect of *MAF1* on *PTEN* was verified by forcing the overexpression of *MAF1* in rBMECs and rAstrocytes. qPCR and western blot analyses showed that the expression levels of *PTEN* were significantly increased by *MAF1* overexpression (Fig. 8A, B), and results of an IHC analysis of MAF1 and PTEN expression were consistent with those obtained by qPCR and western blotting (Fig. 8D). These findings further confirmed the positive regulatory relationship between *MAF1* and *PTEN*. The levels of p-AKT and p-mTOR expression were significantly decreased in the model-S + MAF1 group when compared with the model-S + vector group (Fig. 8C), indicating an inhibitory effect of MAF1 on the AKT/mTOR signaling pathway. The regulation of RIG-I and IRF3 was inhibited by overexpression of *MAF1* (Fig. 8E). Overexpression of *MAF1* promoted the expression of apoptosis-related protein caspase-3, indicating a promotive effect of MAF1 on apoptosis. The autophagy-related proteins p62 and LC3B were significantly inhibited in the model-S + MAF1 group when compared with the model-S + vector group, indicating that overexpression of *MAF1* restored an average level of autophagy level. As shown in Fig. 8F and G**,** IFNβ levels were significantly increased by overexpression of MAF1. When compared with the model-S + vector group, cellular activity was dramatically increased in the model-S + MAF1 group (Fig. 8H), indicating a promotive effect of MAF1 on cell activity. In addition, cell apoptosis was significantly inhibited by* MAF1* (Fig. 8I). As shown in Fig. 8J, autophagy levels were decreased in the model-S + vector group but then recovered to the average level in the model-S + MAF1 group. Recovery of autophagy activity plays an essential role in clearing damaged mitochondria, thus protecting cells. In summary, overexpression of *MAF1* promoted *PTEN* expression, increased cellular activity, decreased cell apoptosis, and normalized autophagy (Fig. 8K).

## Discussion

We proposed a hypothesis for how *MAF1* regulates BBB injury in SAE, and our in vitro and in vivo experiments confirmed the hypothesis. In summary, *CUL2* regulates MAF1 ubiquitination and controls its stability and subsequent Pol III-dependent transcription, thus altering a cell’s sensitivity to apoptosis and causing BBB injury and SAE. The regulatory loop PTEN/AKT/FOXO1/MAF1 regulates Pol III-dependent transcription and plays essential roles in controlling BBB cell activity, cell apoptosis, and autophagy.

Some discrepancies emerged between the present study and our previous study [8]. Our present study confirmed that *MAF1* was expressed at significantly lower levels in four regions of the rat brain in SAE. This low expression, which was stimulated by *CUL2*-mediated ubiquitination, weakened the RIG-I/IRF3 signaling pathway, enhanced cell apoptosis, and recovered normal autophagy. Our previous study demonstrated that *MAF1* also inhibited cell apoptosis, while the levels of *MAF1* expression were increased in lipopolysaccharide-treated BBB cells, which might be owing to the following reasons. First, the in vitro models employed in the two studies were different, with one being cultured with lipopolysaccharide and the other being stimulated by serum. Second, *MAF1* expression was only measured at the protein level, rather than at the mRNA level, in our previous study [8]. In our present study, the levels of *MAF1* expression were not only assessed at the mRNA level, but also at the protein level by western blotting and IHC in different tissues, including myocardium, liver, kidney, and lung. Chen et al. [26] revealed a neuroprotective effect of *MAF1* knockdown on the survival of retinal ganglion cells after injury and suggested a possible therapeutic strategy for traumatic optic neuropathy. Tsang et al. [27] reported that *MAF1* is upregulated and activated in neurons of the peri-infarct cortex in the cerebral hemisphere and functions as an intrinsic suppressor of spontaneous neural repair and functional recovery after ischemic stroke. Wang et al. [28] demonstrated that *MAF1* expression was decreased in a stroke model, and that miR-122 improves the outcome of acute ischemic stroke by targeting *MAF1*. A possible explanation for the varied trends in *MAF1* expression is that *MAF1* may have increased compensation in different cells, and the increase in compensation may be related to the damage time; however, this theory requires further study. Although the levels of *MAF1* expression have varied in other studies, those studies all demonstrated a protective role of *MAF1* in injury models, which is consistent with our present study.

In our present study, we confirmed that knockdown of *CUL2* stimulated *MAF1* expression, attenuated the RIG-I/IRF3 signaling pathway, enhanced cell apoptosis, and recovered normal autophagy. It is well known that *MAF1* is an essential mediator of diverse signals that repress Pol III transcription [29]. Activated Pol III promotes IFNβ production and apoptosis [30, 31]. The Pol II subunit Rpb9 activates ATG1 transcription and autophagy. At the same time, the role played by Pol III in autophagy has rarely been studied. In addition, a report suggested that* RIG-I* plays an important role in dsDNA-induced innate immune activation in human BMECs [32]. *RIG-I* could activate the mitochondrial localization protein IRF3 and induce caspase family protein-dependent apoptosis [33]. Yin et al. identified PTENα, an N-terminally extended form of PTEN, as an RNA-binding protein that blocks RIG-I activation to prevent viral inflammation [34]. The RIG-I-activated signaling pathway may be involved in the apoptosis of blood–brain barrier cells in sepsis. Our present study demonstrated that the shCUL2 induced accumulation of MAF1 normalized the autophagy in SAE. Moreover, this study is the first to reveal the role of *CUL2*/*MAF1* in SAE.

Our study also confirmed the role played by PTEN/AKT/mTOR in BBB damage that occurs in SAE. The experiments showed that overexpression of *PTEN* inhibited IFNβ expression, increased cellular activity, decreased cell apoptosis, and normalized autophagy. Moreover, our study revealed that the AKT/mTOR signaling pathway regulates *MAF1* transcription, inhibits cell activity, and promotes cell apoptosis. Chen et al. [26] demonstrated that *MAF1* has adverse effects on learning and memory, as it inhibits dendritic morphogenesis and the growth of dendritic spines via AKT/mTOR signaling and increasing *PTEN* expression. Although that study and our present study shared the same regulatory mechanism, the studies showed opposite results. In a liver cancer study, *MAF1* was reported to suppress AKT/mTOR signaling by activating *PTEN* transcription and inhibiting cell cycle progression [22]. As previously mentioned, *MAF1* is a newly identified target of *PTEN* and links RNA with lipid metabolism [35]. *MAF1* paradoxically inhibits AKT/mTOR signaling and suppresses cellular growth via a feed-forward loop involving *PTEN* [20]. Therefore, the PTEN/AKT/mTOR/MAF1 axis might play multiple roles in different diseases. Because the PTEN/AKT/mTOR/MAF1 axis has only been studied in SAE, additional comprehensive studies are required.

mTOR is the downstream substrate of AKT, and mTOR activation by AKT inhibits autophagy. Autophagy may exert a protective effect in sepsis-induced myocardial dysfunction [36]. We found that overexpression of *PTEN* normalized autophagy. Inhibition of astrocyte autophagy exacerbates cognitive impairment in sepsis-associated encephalopathy [37]. Furthermore, Qin et al. found that autophagy-mediated regulation of microglial activation contributes to the occurrence and development of SAE [38]. Shi et al. [39] demonstrated that valproic acid attenuates sepsis-induced myocardial dysfunction in rats by accelerating autophagy via the PTEN/AKT/mTOR pathway. Sang et al. [40] also revealed roles played by autophagy and the PTEN/AKT/mTOR pathway, and demonstrated that miR‑214 ameliorates sepsis‑induced acute kidney injury via PTEN/AKT/mTOR regulated autophagy. Therefore, our present study confirmed that PTEN/AKT/mTOR regulates cell activity, apoptosis, and autophagy in BBB injuries caused by sepsis. *PTEN* was reported to participate in sepsis-related diseases via various regulatory mechanisms [41, 42]. For example, lncRNA NEAT1 aggravates sepsis-induced lung injuries by regulating the miR-27a/PTEN axis [43], and miRNA-186 improves sepsis-induced renal injuries via the PTEN/PI3K/AKT/P53 pathway [42]. Our present study revealed a novel regulatory effect of PTEN/AKT/mTOR on SAE.

*FOXO1*, a regulator of endothelial cell proliferation, can accelerate cell apoptosis. Reports have suggested that *FOXO1* levels are increased in the skeletal muscles of sepsis animal models [44, 45]. *FOXO1* also plays important roles in sepsis-associated diseases [46, 47]. Our present study verified the existence of a PTEN/AKT/FOXO1/MAF1 regulatory loop. Studies have shown that microRNAs play essential roles in regulating the PTEN/AKT/FOXO1 axis and especially in regulating cancers. For example, Shen et al. [48] showed that miRNA modulates the PTEN/AKT/FOXO1 pathway to promote the resistance of breast cancer cells to adriamycin. A decreased level of ARHGAP15 expression promotes the development of colorectal cancer via the PTEN/AKT/FOXO1 axis [49]. The PTEN/AKT/FOXO1 pathway is also regulated by microRNA-181a to suppress the proliferation and invasion and promote the apoptosis of cervical cancer cells [50]. In recent years, MAF1 has been found to regulate PTEN/AKT/FOXO1. For example, Liu et al. [24] revealed the existence of this regulatory loop in the dengue virus. Those investigators concluded that PTEN lipid phosphatase activity decreased the size and numbers of cellular lipid droplets via AKT/FoxO1/MAF1 signaling, which enhanced dengue virus replication and virus production, together with autophagy. Palian et al. [21] demonstrated that regulation of MAF1/PTEN/PI3K/AKT/FOXO1 is biologically relevant, as diet-induced PI3K activation reduces *MAF1* expression. Our study is the first to analyze how PTEN/AKT/FOXO1/MAF1 affects a BBB injury caused by SAE. After confirming the regulatory loop, we further analyzed how that loop functions in SAE. We found that interference with *FOXO1* inhibited *MAF1* expression, attenuated the RIG-I/IRF3 signaling pathway, inhibited cell activity, and enhanced cell apoptosis in SAE. Therefore, a third regulatory loop was also confirmed in our present study.

In the final section of the study, we further examined how *CUL2* affected the feedback mechanism of PTEN/AKT/FOXO1/MAF1. We found that *CUL2* influenced the expression level of *MAF1*, which subsequently influenced the PTEN/AKT/FOXO1/MAF1 regulatory loop, and thus controlled Pol III activity and BBB cell apoptosis in SAE. In our present study, we explored the molecular mechanism by which *MAF1* regulates BBB injuries in SAE. We identified three regulatory pathways involved in a BBB injury caused by SAE, and those pathways centered on MAF1.

## Conclusions

*CUL2* regulates MAF1 ubiquitination and controls its stability and subsequent Pol III-dependent transcription, thus promoting BBB cell apoptosis. The regulatory loop PTEN/AKT/FOXO1/MAF1 also regulates Pol III-dependent gene transcription and plays essential roles in BBB cell apoptosis and autophagy. The molecular mechanism of *MAF1* in SAE, as elucidated in our present study, has never been reported before. Therefore, additional verification studies are required.

## Supplementary Information

Supplementary Material 1: Figure S1 Differential gene expression in the brains of septic rats and control rats. Three sepsis rats and three control rats’ brain tissues were applied for transcriptome high-throughput sequencing. (A, B) Volcano and heat maps of differentially expressed genes between Control group and Sepsis group. (C) Top 10 down regulated genes and top 10 up regulated genes. (D, E) Gene enrichment analysis with Gene Ontology (GO) and Kyoto Encyclopedia of Genes and Genomes (KEGG) database. (F) Detection of *MAF1* expression levels in different regions of rat brain tissue using immunofluorescence assay.

Supplementary Material 2: Figure S2 *MAF1* was expressed at low levels in the sepsis in vitro model. (A) Flow cytometry analyses of CD31 and GFAP levels in rBMECs and rAstrocytes. (B) Western blot analyses of CD31 and GFAP expression in rBMECs and rAstrocytes. (C) CCK-8 analyses of rBMECs and rAstrocytes cellular activity following treatment with serum isolated from Sham and model rats. (D-F) qPCR and western blot studies evaluating *MAF1* expression levels in the model-S and sham-S groups. (K) The rBMECs and rAstrocytes were mixed cultured in upper chamber and rat neuronal cells were cultured in lower chamber. After the upper layer cells form a membrane, the serum from control and sepsis model rats was added into upper chamber. (G) Evans blue marked BSA (EB-BSA, 600 ug/ml) was added into upper chamber and after 24h the EB-BSA in lower chamber was measured. (H) The transendothelial electrical resistance. (I) the concentration of IFNβ in the lower chamber. (J) Serum stimulation for 24 hours, the activity of neuronal cells in the lower chamber. ***P < 0.001.

Supplementary Material 3: Figure S3 Expression of *CUL2* and *MAF1* affected membrane permeability. After intervention of gene expression, the rBMECs and rAstrocytes were mixed cultured upper chamber and rat neuronal cells were cultured in lower chamber. (A, E) Evans blue marked BSA (EB-BSA, 600 ug/ml) was added into upper chamber and after 24h the EB-BSA in lower chamber was measured. (B, F) The transendothelial electrical resistance. (C, G) the concentration of IFNβ in the lower chamber. (D, H) Serum stimulation for 24 hours, the activity of neuronal cells in the lower chamber. ***P < 0.001.

## Data Availability

Data will be made available on reasonable request.

## Abbreviations

BBB: Blood–brain barrier
SAE: Sepsis-associated encephalopathy
CLP: Cecum ligation and puncture
rBMECs: Rat brain microvascular endothelial cells
rAstrocytes: Rat brain astrocyte
MAF1: MAF1 homolog, a negative RNA polymerase III
NF-kB: Nuclear factor kappa B
NLRP3: NOD-like receptor family, pyrin domain containing 3
CUL2: E3 ubiquitin ligase Cullin 2
RIG-I: Retinoic acid-induced gene I
IRF3: Interferon regulatory factor 3
HIF: Hypoxia-inducible factor α
PTEN: Phosphatase and tensin homolog
AKT: Serine/threonine kinase
mTOR: Mechanistic target of rapamycin kinase
FOXO1: Forkhead box O1
CHX: Cycloheximide
