# Supplementary figures and images for: Feedback loop centered on MAF1 reduces blood–brain barrier damage in sepsis-associated encephalopathy
Source: Cell Mol Biol Lett. 2025 Jan 20;30:8. doi: 10.1186/s11658-025-00686-x (PMC11744841; doi:10.1186/s11658-025-00686-x)

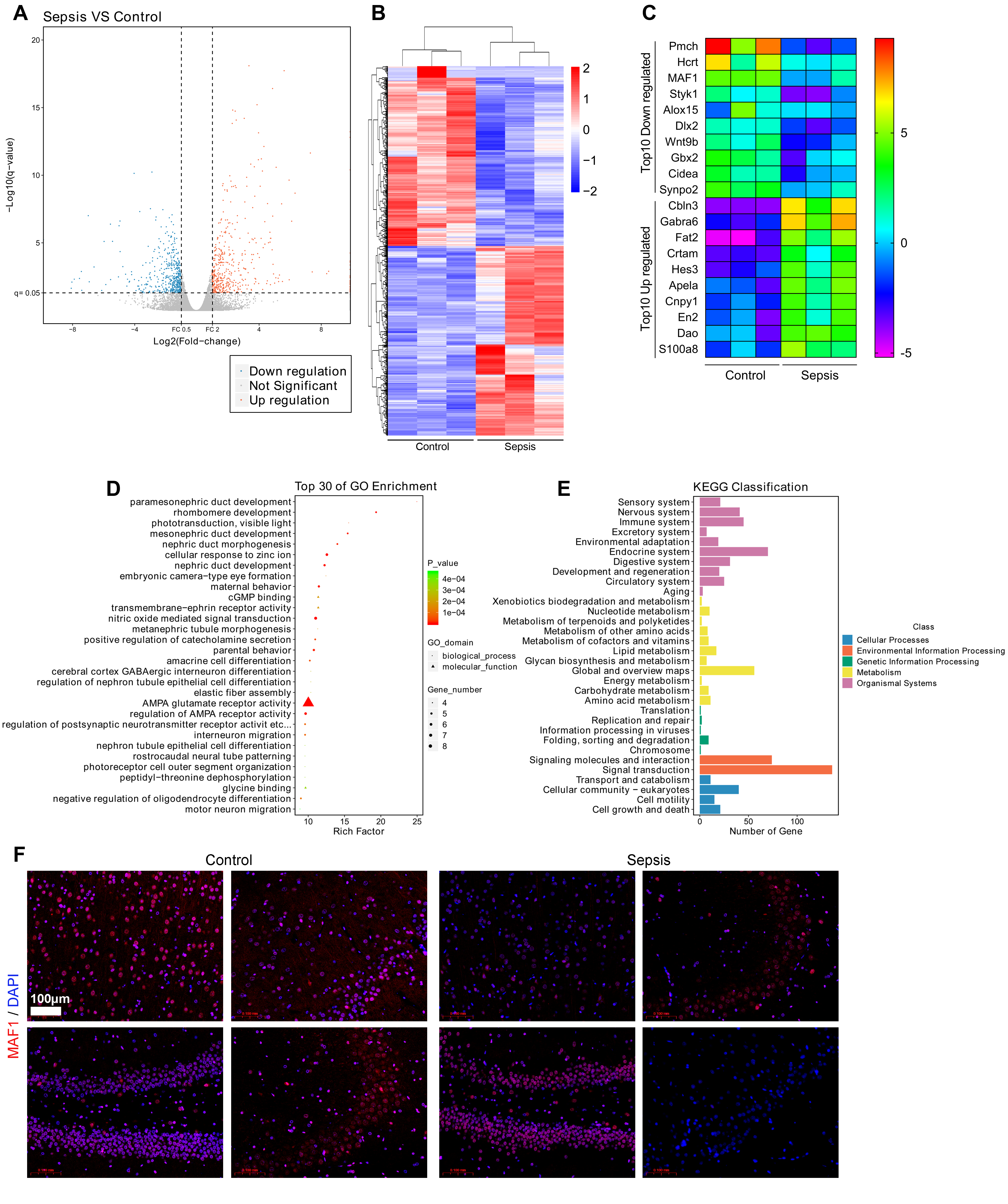

Supplement: Supplementary file 1 — Supplementary Material 1: Figure S1 Differential gene expression in the brains of septic rats and control rats. Three sepsis rats and three control rats’ brain tissues were applied for transcriptome high-throughput sequencing. (A, B) Volcano and heat maps of differentially expressed genes between Control group and Sepsis group. (C) Top 10 down regulated genes and top 10 up regulated genes. (D, E) Gene enrichment analysis with Gene Ontology (GO) and Kyoto Encyclopedia of Genes and Genomes (KEGG) database. (F) Detection of MAF1 expression levels in different regions of rat brain tissue using immunofluorescence assay. [file 11658_2025_686_MOESM1_ESM.tif]

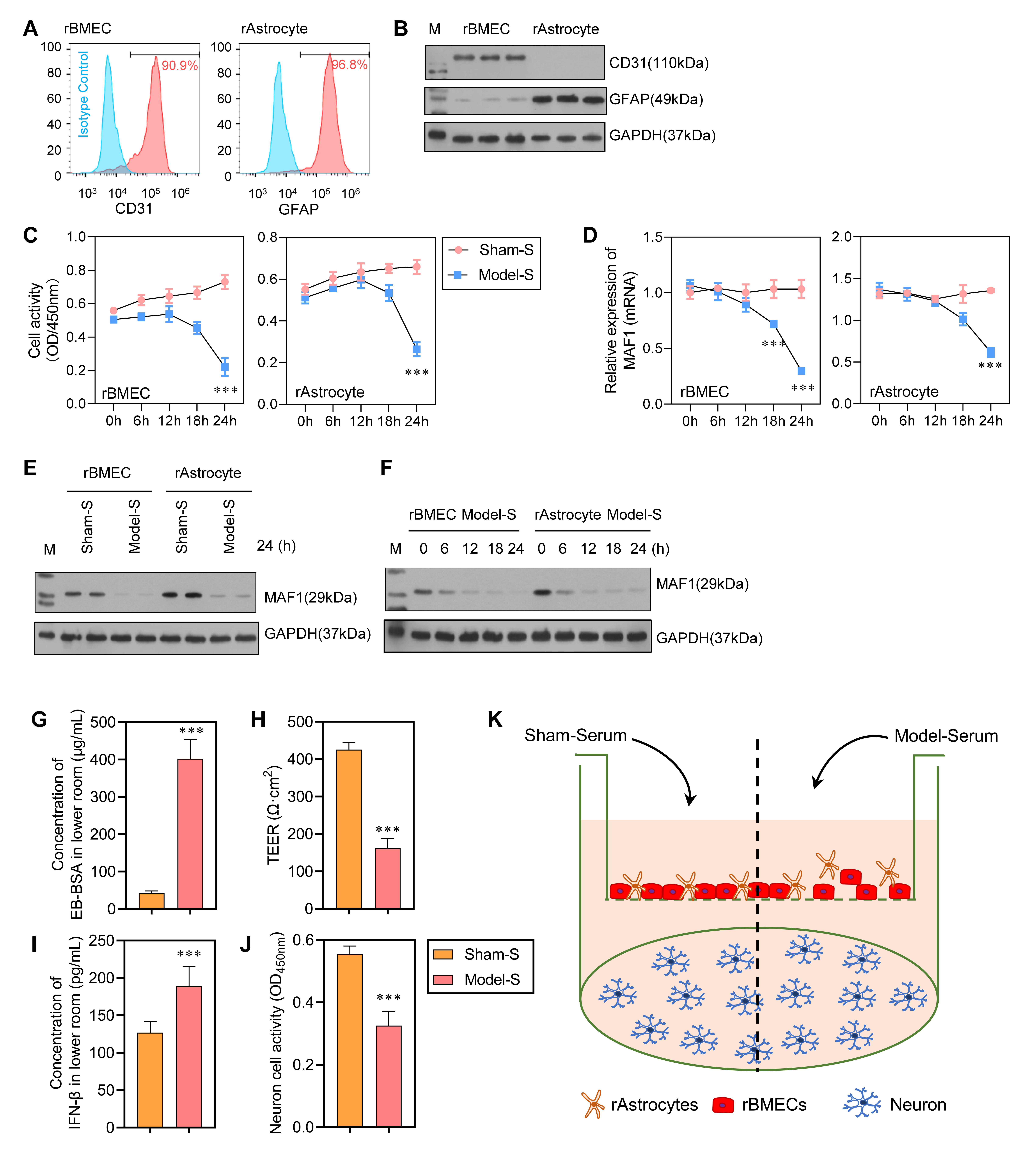

Supplement: Supplementary file 2 — Supplementary Material 2: Figure S2 MAF1 was expressed at low levels in the sepsis in vitro model. (A) Flow cytometry analyses of CD31 and GFAP levels in rBMECs and rAstrocytes. (B) Western blot analyses of CD31 and GFAP expression in rBMECs and rAstrocytes. (C) CCK-8 analyses of rBMECs and rAstrocytes cellular activity following treatment with serum isolated from Sham and model rats. (D-F) qPCR and western blot studies evaluating MAF1 expression levels in the model-S and sham-S groups. (K) The rBMECs and rAstrocytes were mixed cultured in upper chamber and rat neuronal cells were cultured in lower chamber. After the upper layer cells form a membrane, the serum from control and sepsis model rats was added into upper chamber. (G) Evans blue marked BSA (EB-BSA, 600 ug/ml) was added into upper chamber and after 24h the EB-BSA in lower chamber was measured. (H) The transendothelial electrical resistance. (I) the concentration of IFNβ in the lower chamber. (J) Serum stimulation for 24 hours, the activity of neuronal cells in the lower chamber. ***P < 0.001. [file 11658_2025_686_MOESM2_ESM.tif]

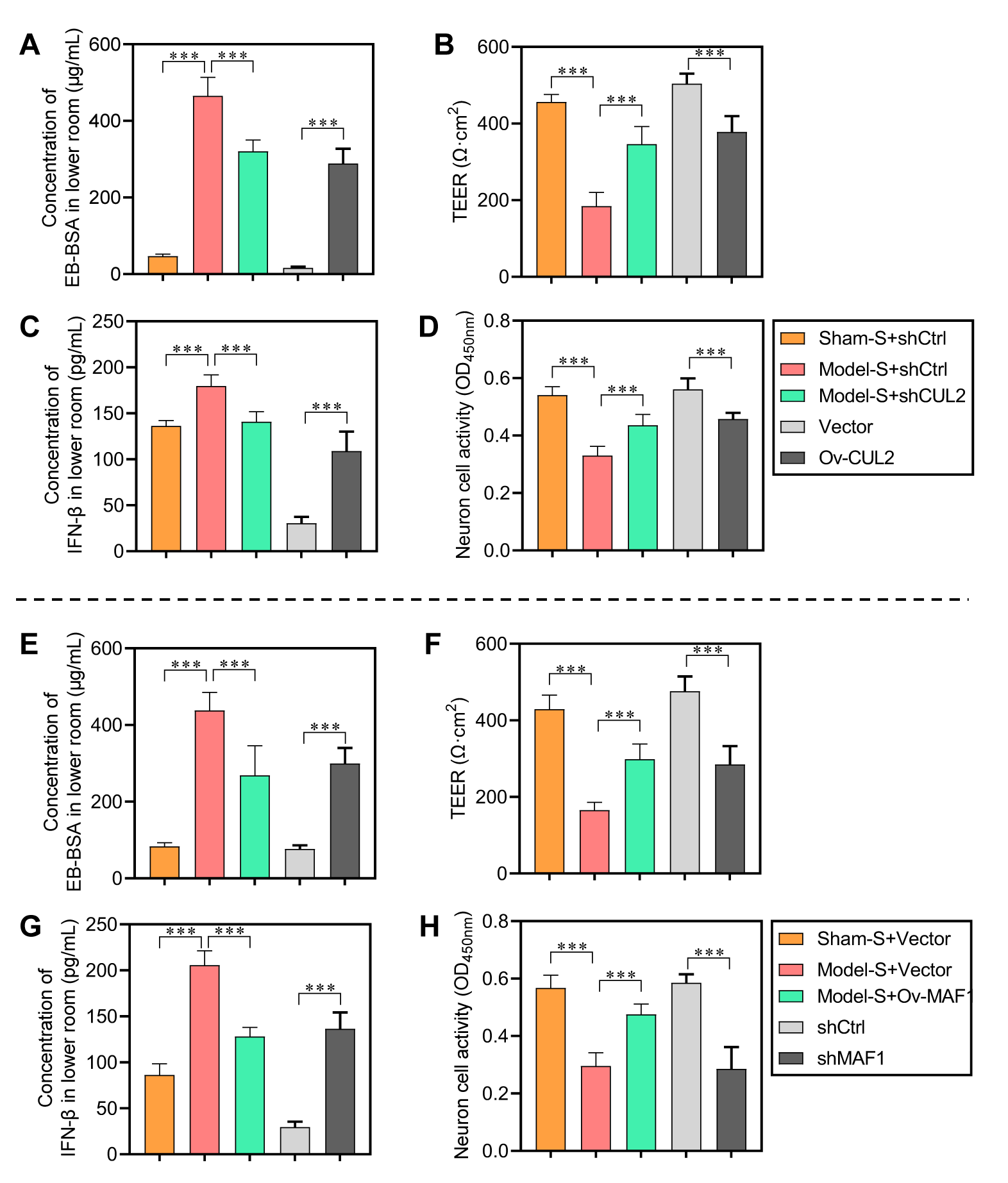

Supplement: Supplementary file 3 — Supplementary Material 3: Figure S3 Expression of CUL2 and MAF1 affected membrane permeability. After intervention of gene expression, the rBMECs and rAstrocytes were mixed cultured upper chamber and rat neuronal cells were cultured in lower chamber. (A, E) Evans blue marked BSA (EB-BSA, 600 ug/ml) was added into upper chamber and after 24h the EB-BSA in lower chamber was measured. (B, F) The transendothelial electrical resistance. (C, G) the concentration of IFNβ in the lower chamber. (D, H) Serum stimulation for 24 hours, the activity of neuronal cells in the lower chamber. ***P < 0.001. [file 11658_2025_686_MOESM3_ESM.tif]
